# High‐density SNP genotyping array for hexaploid wheat and its secondary and tertiary gene pool

**DOI:** 10.1111/pbi.12485

**Published:** 2015-10-15

**Authors:** Mark O. Winfield, Alexandra M. Allen, Amanda J. Burridge, Gary L. A. Barker, Harriet R. Benbow, Paul A. Wilkinson, Jane Coghill, Christy Waterfall, Alessandro Davassi, Geoff Scopes, Ali Pirani, Teresa Webster, Fiona Brew, Claire Bloor, Julie King, Claire West, Simon Griffiths, Ian King, Alison R. Bentley, Keith J. Edwards

**Affiliations:** ^1^ Life SciencesUniversity of BristolBristolUK; ^2^ Affymetrix UK LtdHigh WycombeUK; ^3^ School of BiosciencesSutton BoningtonLeicestershireUK; ^4^ John Innes CentreNorwich Research ParkNorwichNorfolkUK; ^5^ The John Bingham LaboratoryNIABCambridgeUK

**Keywords:** wheat, secondary and tertiary gene pools, wheat progenitors, next‐generation sequencing, genotyping array, single nucleotide polymorphism

## Abstract

In wheat, a lack of genetic diversity between breeding lines has been recognized as a significant block to future yield increases. Species belonging to bread wheat's secondary and tertiary gene pools harbour a much greater level of genetic variability, and are an important source of genes to broaden its genetic base. Introgression of novel genes from progenitors and related species has been widely employed to improve the agronomic characteristics of hexaploid wheat, but this approach has been hampered by a lack of markers that can be used to track introduced chromosome segments. Here, we describe the identification of a large number of single nucleotide polymorphisms that can be used to genotype hexaploid wheat and to identify and track introgressions from a variety of sources. We have validated these markers using an ultra‐high‐density Axiom^®^ genotyping array to characterize a range of diploid, tetraploid and hexaploid wheat accessions and wheat relatives. To facilitate the use of these, both the markers and the associated sequence and genotype information have been made available through an interactive web site.

## Introduction

Bread wheat (*Triticum aestivum*) is an allohexaploid crop derived from the hybridization of diploid *Aegilops tauschii* with tetraploid wild emmer, *Triticum turgidum* ssp. *dicoccoides* (Dubcovsky and Dvorak, 2007; Matsuoka, 2011; Shewry, 2009). This hybridization, subsequent domestication and inbreeding have reduced genetic diversity in cultivated wheat compared with its wild ancestors (Haudry *et al*., 2007; Tanksley and McCouch, 1997). The lack of genetic diversity is a major issue for wheat breeders and limits their ability to produce new varieties (Roussel *et al*., 2004; White *et al*., 2008). Hybridization of wheat with wild relatives, resulting in so‐called alien introgression, has been used on numerous occasions to introduce novel diversity into bread wheat's gene pool (Chen *et al*., 2012; Molnár‐Láng *et al*., 2014). However, introgression of ‘alien’ DNA into the wheat genome inevitably leads to the introduction of undesirable traits as genes linked to the target gene are introduced along with it, so‐called linkage drag (Klindworth *et al*., 2013). The negative impact of linkage drag can be minimized by reducing the size of the introgressed fragment to the minimum necessary to retain the desired phenotype (Wulff and Moscou, 2014). This can be achieved through repeated backcrossing to the elite parent but is often a lengthy process (Qi *et al*., 2007). Until recently, evaluation of introgressions has been conducted using manually intensive cytogenetic techniques which cannot be readily applied to a large number of samples (Friebe *et al*., 1991, 1996; Lukaszewski *et al*., 2005). Molecular markers, on the other hand, which can be adapted for high sample throughput, enable the rapid and cost‐effective characterization of introgressions (Thomson, 2014).

The use of molecular markers, such as single nucleotide polymorphisms (SNPs), is now common place in the genotyping of wheat (Akhunov *et al*., 2009; van Poecke *et al*., 2013). The uptake of SNP markers has recently been accelerated by the use of both KASP assays (Allen *et al*., 2011; LGC, Herts, UK) and the development of a high‐density iSelect array (Wang *et al*., 2014; Illumina, San Diego, CA). However, while the development of the current hexaploid SNP resources is welcome, the majority of SNP markers developed to date are not suitable for use in wide crosses. The high level of sequence polymorphism between hexaploid wheat and its wild relatives makes it difficult to design polymerase chain reaction (PCR) primers for array‐based probes. Recently, Tiwari *et al*. (2014) overcame this problem by sequencing flow‐sorted wheat chromosomes to identify SNPs on the homoeologous group five chromosomes in a cross between Chinese Spring and *Aegilops geniculata*. Their work, however, also highlighted the high cost and attrition level of developing large numbers of validated SNP markers. To overcome this problem, Wang *et al*. (2014) used an array‐based platform to examine and validate over 81 000 putative SNPs in both tetraploid and hexaploid wheat, and were able to validate 56 388. SNPs derived from *Ae. tauschii*, the D genome donor of hexaploid wheat, were also included on their array, and of the approximately 4400 SNPs derived from this species, 796 (18%) were also polymorphic in a range of hexaploid wheat accessions (Wang *et al*., 2014).

We recently reported the use of a sequence capture targeted re‐sequencing approach to characterize a significant proportion of the wheat exome (Winfield *et al*., 2012), which was then used to identify large numbers of exome‐specific SNPs (Allen *et al*., 2013). Here, we have extended this procedure to include the equivalent exome‐captured sequences from a range of species, including members of the secondary and tertiary gene pool, that are a potential source of novel alleles suitable for introgression into the hexaploid genome. We have analysed the resulting captured sequences to identify a large number of putative SNPs between different varieties of hexaploid wheat and between hexaploid wheat and related species, including its putative progenitor species (*Ae. tauschii*,* Aegilops speltoides* and *Triticum urartu*) and various wild relatives. To carry out a large‐scale validation of the putative SNP markers, we used the Axiom^®^ high‐density genotyping platform (Affymetrix Inc., Santa Clara, CA). The SNP markers and the Axiom^®^ genotyping array described here have resulted in the generation of a large number of validated varietal and species‐specific SNPs which can be used to monitor and map introgressions within the hexaploid wheat genome.

## Results

### SNP discovery

Using a wheat NimbleGen array (Winfield *et al*., 2012) to direct the capture and targeted re‐sequencing of the wheat exome, we generated ~900 million sequences from 43 bread wheat accessions and wheat relatives. These included 14 diploid species including A, B and D genome progenitors as well as representatives of E, J, R and T genomes, five tetraploids (AB and AG), 23 hexaploids (ABD and SJJ) and one decaploid (JJJJ^s^J^s^) (Table S1). Of the sequences generated, 344.5 million (38%) could be mapped back to sequences on the array.

To identify polymorphic sequences within the species used, we used the SNP discovery pipeline and experimental procedures described by Winfield *et al*. (2012) to obtain 921 705 putative varietal SNPs from the mapped sequences (this data set may be downloaded from the CerealsDB web site; http://www.cerealsdb.uk.net/cerealgenomics/CerealsDB/Excel/PutativeSNPs.csv).

Putative SNPs, together with their flanking sequences, were processed using the Affymetrix design protocol for the Axiom^®^ platform to generate 819 571 putative SNP probes (Axiom^®^ HD Wheat Genotyping Array; this data set may be downloaded from the CerealsDB web site: http://www.cerealsdb.uk.net/cerealgenomics/CerealsDB/Excel/axiom820Data.txt.zip). Of the 819 571 SNPs, 528 961 (64.5%) were transitions and 290 610 (35.5%) were transversions. This compares with 72% and 28%, respectively, observed by Wang *et al*. (2014).

The NimbleGen array contained 132 606 repeat‐masked expressed sequence tags obtained from hexaploid wheat (Winfield *et al*., 2012). Of these features, 81 132 (61%) were found to have at least one SNP with 64 937 (49%) features having three or more SNPs.

As chromosome location is an important consideration when selecting SNPs for genotyping projects, we describe the location of the SNP probes with reference to the recently published IWGSC survey sequences (The International Wheat Genome sequencing Consortium, 2014). We used the ‘Exonerate’ program (Slater and Birney, 2005) to align the SNP probes to the IWGSC survey sequences. We were able to align 547 167 (66.8%) of the SNP probes to 60 841 of the 10 776 707 IWGSC survey sequence contigs (Table 1). Of these, 491 792 (60% of the probes on the array, or 89.9% of the aligned markers) had an unambiguous, single top hit. For all other sequences, it was not possible to determine which homoeologous chromosome was the source of the original SNP probe as probes aligned with equal scores to two or more IWGSC sequences.

**Table 1:** Distribution of SNP probes across the twenty‐one hexaploid wheat chromosomes as determined using the Exonerate software

Chromosome	Contigs per chromosome		SNPs assigned		SNP containing contigs	
	Number	Percentage	Number	Percentage	Number	Percentage
1A	385 164	3.57	21 708	4.41	2577	4.24
1B	380 769	3.53	20 955	4.26	2592	4.26
1D	418 941	3.89	22 831	4.64	2495	4.10
2A	586 072	5.44	28 131	5.72	3322	5.46
2B	610 231	5.66	29 349	5.97	3688	6.06
2D	753 346	6.99	29 467	5.99	3933	6.46
3A	546 152	5.07	20 588	4.19	2842	4.67
3B	546 922	5.08	28 508	5.80	3357	5.52
3D	641 702	5.95	20 763	4.22	3078	5.06
4A	663 964	6.16	22 968	4.67	2975	4.89
4B	591 798	5.49	20 418	4.15	2769	4.55
4D	572 506	5.31	22 277	4.53	2811	4.62
5A	586 203	5.44	20 387	4.15	2567	4.22
5B	573 553	5.32	29 635	6.03	3668	6.03
5D	371 504	3.45	29 203	5.94	2876	4.73
6A	456 255	4.23	19 395	3.94	2306	3.79
6B	418 338	3.88	17 385	3.54	2454	4.03
6D	292 347	2.71	22 473	4.57	2191	3.60
7A	495 959	4.60	19 857	4.04	2702	4.44
7B	507 514	4.71	18 679	3.80	2663	4.38
7D	377 467	3.50	26 815	5.45	2975	4.89
Total	10 776 707	100.00	491 792	100.00	60 841	100.00

**Figure 1:**
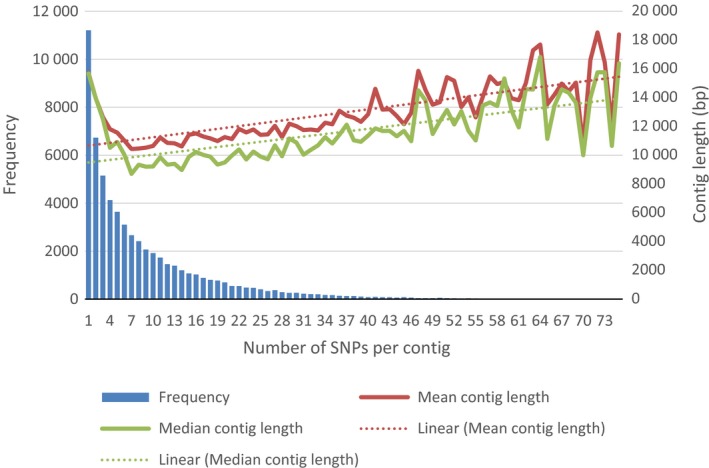
Single nucleotide polymorphisms (SNP) probe distribution across the hexaploid genome. Distribution of SNP‐probes per IWGSC contigs compared to contig length. The number of SNP‐probes per IWGSC was determined using ‘Exonerate’. For each SNPs per contig grouping the mean (red line) or median (green line) size of the contigs in base pairs (bp) was determined by standard means as was the linear regression (dotted line) of the mean contig length.

Examination of the genome distribution of the 60 841 IWGSC contigs containing the 547 167 SNP probes indicated that they were evenly distributed across the 21 hexaploid wheat chromosomes (Table 1). Further examination of the SNP probe distribution across the 60 841 IWGSC contigs suggested that while 11 210 contigs contained a single SNP probe, the remaining contigs aligned to multiple probes (Figure 1).

### SNP validation

The Axiom^®^ HD Wheat Genotyping Array (hereafter referred to as the Axiom^®^ Array) was used to screen genomic DNA prepared from 475 accessions (listed in Table S2). These included 108 elite hexaploid accessions of which 48 were suggested by a number of commercial wheat breeders, 27 hexaploid accessions from the Watkins collection (Burt *et al*., 2014; Miller *et al*., 2001), eight *T. turgidum* accessions and 24 wheat relatives including *T. urartu*,* Ae. speltoides* and *Ae. tauschii* (A, B and D genome progenitors, respectively). We included twenty lines from the Chinese Spring nullisomic collection (Devos *et al*., 1999) and 32 accessions from the Kansas deletion line collection (Endo and Gill, 1996) to allow us to physically assign SNP probes to chromosomes. We also included individuals from the Avalon × Cadenza, Savannah × Rialto (Limagrain, UK) and Synthetic × Opata (Sorrels *et al*., 2011) mapping populations.

Genotype calls were generated as described in Experimental procedures. The sample call rate ranged from 80.1% to 99.6% with an average of 98.4% for the 475 accessions. The average call rate varied depending upon the ploidy and relationship of the accessions screened (Table S3). The lowest call rates were obtained for the wheat relatives with an average of 85.8%. The 14 *Ae. tauschii* accessions had a higher average call rate (92.3%) than either of the other two representatives of the A and B genomes; *T. urartu* (83.2%) and *Ae. speltoides* (85.4%). For the 819 571 SNP probes on the array, the call rate ranged from 4.4% to 100% with an average of 98.4%. Of these, 765 359 (93.4%) had a call rate of greater than 95%.

**Figure 2:**
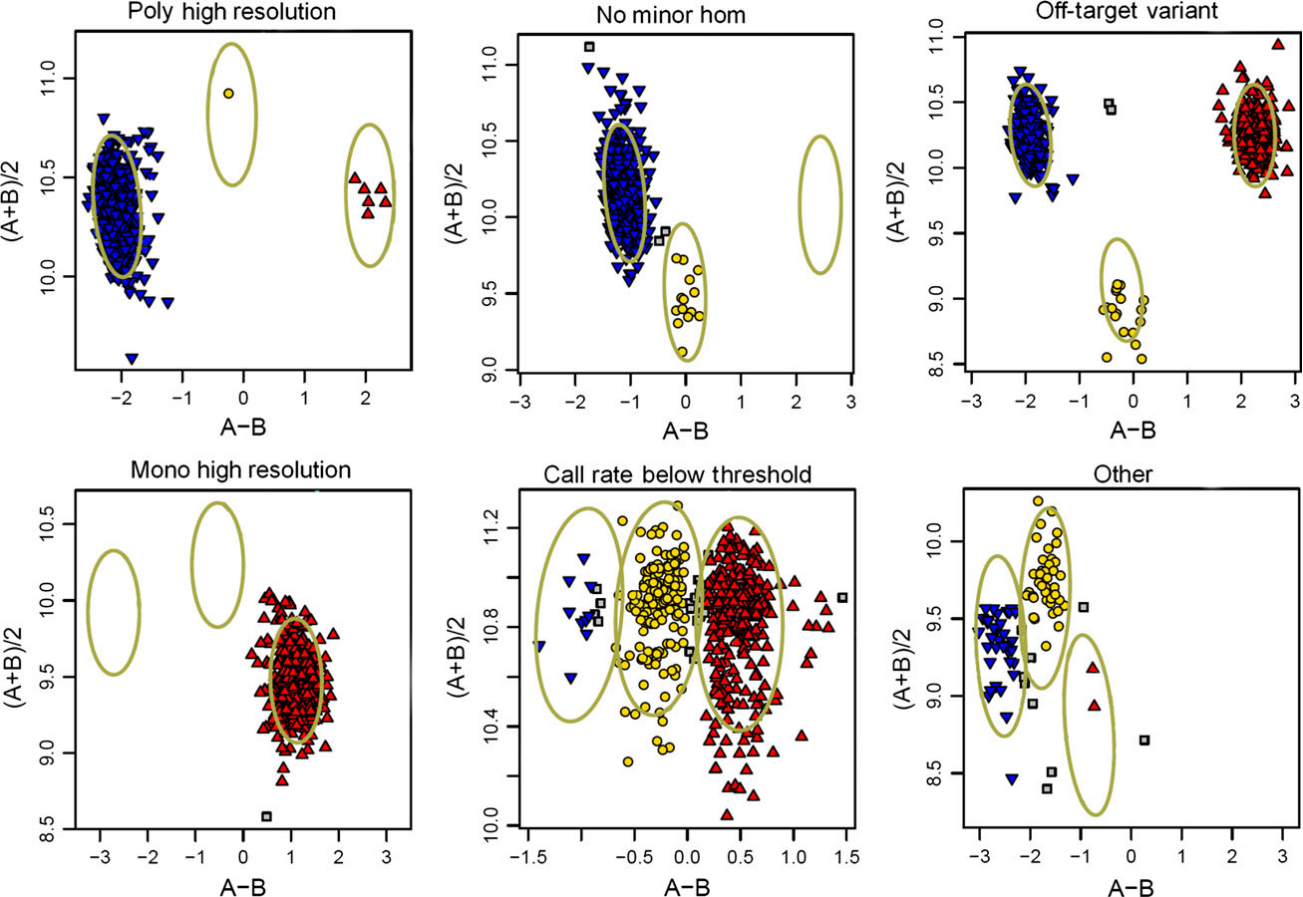
Examples of the six probe calling categories: (a) Poly High Resolution; (b) No Minor Hom; (c), Off‐Target Variants (OTV); (d) Mono High Resolution; (e) Call Rate Below Threshold; and (f) Other.

The scores for the probes were classified into one of six categories according to the cluster pattern produced by the Affymetrix software (Figure 2); (i) Poly High Resolution (PHR) (53 569; 6.5%); (ii) No Minor Homozy**gote (NMH) (449 941; 54.9%); (iii) Off‐Target Variants (OTV) (42 789; 5.2%); (iv) Mono High Resolution (MHR) (144 320; 17.6%); (v) Call Rate Below Threshold (CRBT) (23 686; 2.9%); and (vi) Other (105 266; 12.8%). Only the first three groups (‘PHR’, ‘NMH’ and ‘OTV’) were considered useful; a total of 546 299 probes (66.7%) fell into one of these categories.

**Figure 3:**
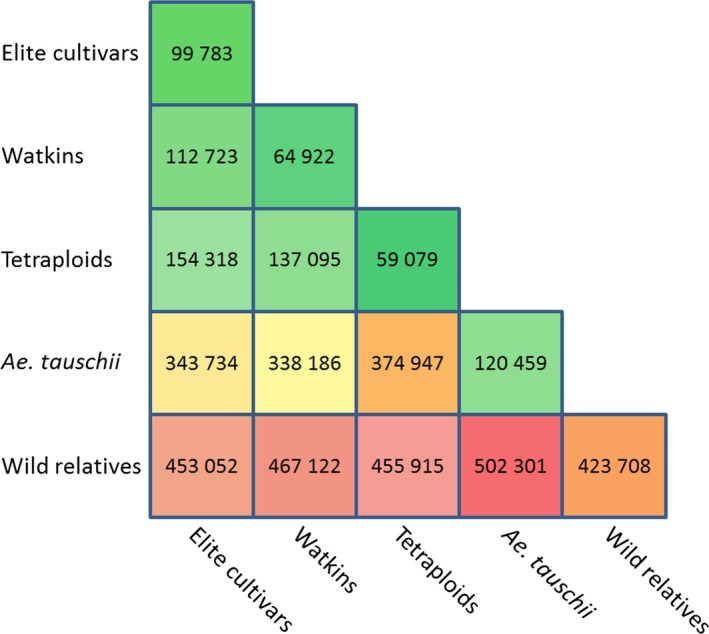
Number of probes categorised as polymorphic and high quality for each of the different comparisons. Box colours highlight the number of polymorphisms within and between groups; green represents low numbers and red high numbers.

The Axiom^®^ Array was designed to genotype hexaploid wheat as well as species from the secondary and tertiary gene pools. To confirm the array's utility, we considered the 546 299 polymorphic SNP probes. Of these, 99 783 were polymorphic between the 108 elite hexaploid wheat varieties, a figure that increased to 112 723 when the Watkins collection was included and to 453 052 when the elite accessions and their relatives and progenitors were considered. The number of polymorphic probes between the different groups is shown in Figure 3. The complete data set for all 475 accessions can be downloaded as a CSV file from the CerealsDB web site (http://www.cerealsdb.uk.net/).

### Physical mapping of the SNP probes

To putatively assign markers to chromosomes, genomic DNA from the eighteen Chinese Spring derived nullisomic/tetrasomic accessions and the 32 Kansas deletion accessions was screened against the array. Through this approach, 161 869 markers (nullisomic/tetrasomic lines) and 127 990 markers (Kansas deletion lines) were physically assigned to a chromosome (Table 2).

**Table 2:** Physical location of the SNP probes. Physical location was determined using either the available nullisomic/tetrasomic lines or the Kansas deletion lines

Chromosome	Exonerate	Nullitetra	Kansas
1A	21 708	5328	3769
1B	20 955	7179	8786
1D	22 831	8736	8150
2A	28 131	1413	2684
2B	29 349	12 814	4454
2D	29 467	10 892	3384
3A	20 588	13 760	11 518
3B	28 508	—–a	4535
3D	20 763	14 790	3717
4A	22 968	9792	–b
4B	20 418	1341	2186
4D	22 277	8485	6735
5A	20 387	11 121	5294
5B	29 635	7863	2616
5D	29 203	6540	4190
6A	19 395	5198	–b
6B	17 385	4209	5581
6D	22 473	8324	–b
7A	19 857	6125	6731
7B	18 679	7037	37 482
7D	26 815	10 922	6178
Total	491 792	161 869	127 990

a We did not include a 3B nullisomic/tetrasomic line so could not map markers to this chromosome.

b Deletion lines for these chromosomes were not included on the array.

### Genetic mapping

The number of polymorphic markers between the parental lines of each populations was 23 740 (Avalon × Cadenza), 21 285 (Savannah × Rialto) and 38 019 (Synthetic W7984 × Opata). Markers with more than 20% missing data were removed before map construction. Markers that had a unique pattern of segregation were also removed. The number remaining for each population was 20 536, 19 683 and 34 513, respectively.

#### Avalon × Cadenza

The 20 536 markers fell into 1447 bins. From each of these bins, one marker was selected as a representative to create a chromosome frame. A chi‐square test of these representatives showed that 157 exhibited significant segregation distortion (*P* < 0.05). These markers were also removed along with the markers in the bin they represented. Thus, there were 1290 markers from which to construct the chromosome frame. Of these, 1286 markers mapped to 21 linkage groups representing the 21 wheat chromosomes, and four markers were unlinked. These four markers, along with the all other markers from the bin for which they were the representative, were also removed. The total map length of this ‘frame’ was 3663 cM with an average chromosome length of 174 cM and one marker every 2.9 cM. Finally, the markers from the initial bins were reintegrated into the map at the same cM position as their representative ‘frame’ markers. The complete map contained 18 942 markers (Table 3 and Tables S4 and S5).

**Table 3:** Genetic location of the SNP probes determined using one of the three mapping populations Avalon × Cadenza (A × C), Savannah × Rialto (S × R) and Synthetic × Opata (S × O). Genetic maps are available in Tables S4 and S5

Chromosome	A × C	S × R	S × O	Consensus
1A	988	1353	1371	2938
1B	2379	885	2306	4303
1D	896	471	979	2077
2A	873	487	1285	2451
2B	1709	2876	2368	5967
2D	506	61	1168	1599
3A	697	630	1144	2083
3B	1051	1037	1787	3207
3D	152	368	2010	2344
4A	905	242	1518	2358
4B	599	225	1240	1756
4D	55	86	703	813
5A	802	1048	1022	2348
5B	1191	985	2138	3844
5D	425	413	1519	2019
6A	1459	1146	1565	3129
6B	1557	1771	1739	4090
6D	143	187	1192	1397
7A	1161	965	1378	2851
7B	1123	559	2110	3251
7D	271	244	1266	1680
Total	18 942	16 039	31 808	56 505

#### Savannah × Rialto

The 19 683 markers fell into 830 bins from each of which a single marker was selected as a representative. A chi‐square test identified 62 markers with significant segregation distortion (*P* < 0.05), and these were removed leaving a core set of 768 markers. A total of 655 markers mapped to 23 linkage groups, while 113 markers were unlinked. These unlinked markers, along with all the markers from the bins they represented, were removed from further analysis. Due to the large genetic distance between markers on the long and short arms of chromosomes 5B and 6D, these were split into two groups, one for the short arm and one for the long arm. The total map length was 2819 cM with an average chromosome length of 136.2 cM and one marker every 4.3 cM. Finally, the markers from the initial bins were reintegrated into the map along with their representative ‘frame’ markers. The complete map contained 16 039 markers (Table 3 and Tables S4 and S5).

#### Synthetic W7984 × Opata

The 34 513 markers fell into one of 2361 bins and one marker was picked to represent each bin. A chi‐square test showed 113 markers with significant segregation distortion (*P* < 0.05) which, once removed, left a core set of 2248 markers. A total of 2167 markers mapped to 21 linkage groups while 81 markers were unlinked. The total map length was 7745 cM with an average of one marker every 3.6 cM and an average chromosome length of 369 cM. After reintegration of binned markers, the map contained 31 808 markers (Table 3 and Tables S4 and S5).

#### Consensus map

In total, we have mapped 56 505 markers to the 21 wheat chromosomes. Of these, 47 069 (83.3%) mapped in only one of the populations, 8588 (15.2%) mapped in two populations and 848 (1.5%) mapped in all three populations. Of the 9436 markers that mapped in more than one of the populations, 729 (7.7%) mapped to different chromosomes on the different maps (Table S6). Of these conflicts, 67.4% were between homoeologous chromosomes, 5.3% were conflicts between chromosomes 5B and 7B, and 5.6% were between chromosomes 4A and 7A. Of the markers in conflict, 67 were mapped in all three of the populations. For these markers, the ‘consensus chromosome’ was assigned based on ‘majority rule’ (if a marker mapped to the same chromosome in two of three maps, this location was used). For the remaining 662 SNPs, 48 were assigned to a consensus chromosome using genotype scores from the nullisomic and Kansas deletion lines and 132 were assigned to a consensus chromosome using information from IWGSC survey sequence contigs. Finally, for the 482 markers that had two map positions (964 chromosome positions), but no physical information, one was chosen at random. The final consensus map with 56 505 markers was 3739 cM in length, with an average of 178 cM per chromosome (Table 3 and Table S5).

### Characterization of hexaploids, progenitors and wheat relatives

**Figure 4:**
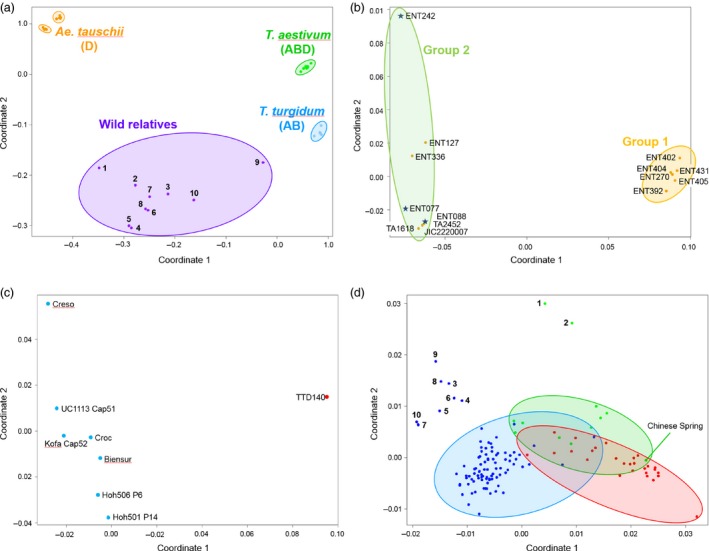
(a) Principal coordinate plot (multidimensional scaling) of all 167 lines (14 D genome, 8 AB genome tetraploids, 10 wild relatives, 108 ABD genome hexaploids and 27 Watkins lines) against 546 299 SNP‐markers. The wild relatives are: 1. *Ae. caudata* (*Ae. markgrafii*; C genome), 2. *Ae. mutica* (syn. *Amblyopyrum muticum*; T genome), 3. *Ae. speltoides* (closest living relative to the B genome progenitor), 4. *S. cereale* (R genome), 5. *Th. bessarabicum* (J genome), 6. *Th. elongatum* (E genome), 7. *Th. intermedium* (JJ ^s^S), 8. *Th. ponticum* (JJJJ ^s^J^s^ genome), 9. *T. timopheevii* (GA genome), 10. *T. urartu* (syn. *T. monococcum* ssp. *aegilopoides*; A genome progenitor). The genomes, ploidy and synonyms of these species are given in Table S1. (b) PCO plot of the putative lines belonging to the D genome progenitor, *Ae. tauschii*. Two distinct clusters are formed; these essentially reflect subspecies (*Ae. tauschii* ssp. *strangulata* or *Ae. tauschii* ssp. *tauschii*) and geographical location of collection. The *strangulata* lines, which are indicated by a blue star, all come from northern Iran. (c) PCO plot of the *T. turgidum* accessions. The first coordinate separates the *T. turgidum* ssp. *dicoccoides* line (red dot) from all the other lines that belong to subspecies *durum*. (d) PCO plot of the hexaploid accessions; blue = winter wheats, green = spring wheats, red = Watkins collection. The numbered lines are those that carry the 1BS/1RS translocation: 1 = Bacanora, 2 = Bobwhite, 3 = Brompton, 4 = Gatsby, 5 = Humber, 6 = Kielder, 7 = Lynx, 8 = Relay, 9 = Rialto, 10 = Savannah. Please note that the accessions Lynx and Savannah (7 and 10, respectively) collocate on the PCO plot.

To date, most genotyping arrays have been designed for use with a single, often diploid, species. Here, we have designed a single array capable of characterizing multiple species with levels of ploidy ranging from diploid, for example *Ae. tauschii* to decaploid, for example *Thinopyrum ponticum* and different genomes with varying degrees of similarity. The relationship between the accessions was determined by calculating a pairwise similarity matrix (Table S7) that was used to perform multidimensional scaling (MDS) and create principal coordinate (PCO) plots. Clear groups were evident (Figure 4a). Tight clusters were produced for the *T. aestivum*,* T. turgidum* and *Ae. tauschii* accessions. The wheat relatives, including *Ae. speltoides* and *T. urartu*, formed a loose cluster. The *Ae. tauschii* accessions, for which there were 120 459 polymorphic probes, fell into two distinct groups. One group (Group 1) contained only subspecies *tauschii,* while the other group (Group 2) contained both subspecies *tauschii* and *strangulata*. All but one of the Group 1 individuals were from China, whereas those in Group 2 had a wide geographic distribution but with the *strangulata* individuals originating from the southern Caspian in Iran or Turkmenistan (Figure 4b). The Axiom^®^ Array was able to separate the *T. turgidum* accessions according to subspecies; ssp. *dicoccoides* accession (TTD140) was clearly distinct from the seven durum wheat accessions (Figure 4c).

**Figure 5:**
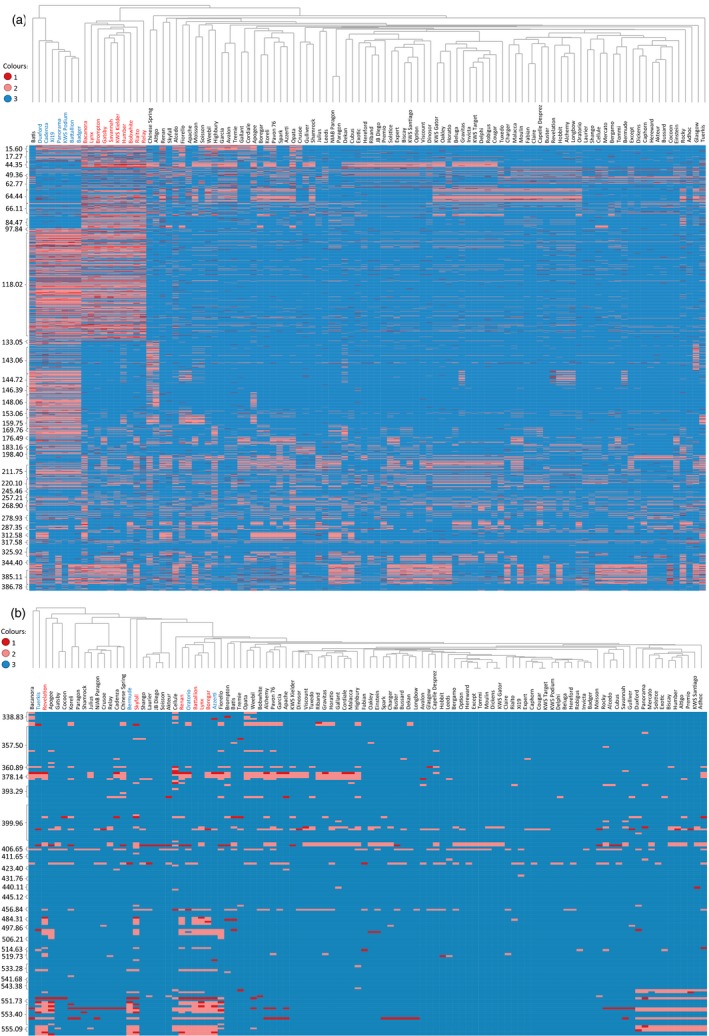
Heatmaps of genotype scores of 104 hexaploid varieties for loci mapped to chromosome (a) 1B and (b) 7DL. The genotypes are organised horizontically by a dendrogram produced using hierarchical cluster analysis and vertically by centimorgan position along the chromosome according to the Synthetic × Opata genetic map. Genotype scores have been coded for each locus as: 1 = least common genotype score; 2 = second most common genotype score and 3 = most common genotype score, and have been coloured according to the legend shown. (a) The heatmap of chromosome 1B shows the distinct haplotypes between those lines carrying the 1RS/1BS substitution (accession names highlighted in red; 0–133 cM) and those lines that do not. This figure also displays the lines belonging to Cadenza derived accessions (accession names highlighted in blue) which have a distinct haplotype on 1B (97.8–198 cM). (b) The heatmap of 7DL highlights accessions carrying *Ae. ventricosa* introgressions (accession names highlighted in red, 456.8–556.8 cM; accession names highlighted in blue, 551.7–556.8 cM).

To confirm that the Axiom^®^ Array was able to dissect the substructure of the hexaploid accessions (elite and Watkins), we examined these in isolation. Two broad groups were evidenced; (i) winter wheats and (ii) spring wheats/Watkins accessions (Figure 4d). Ten accessions, eight winter and two spring, were separated from their main groups; these accessions carry the rye 1RS translocation. To examine this further, we used the 2306 Synthetic × Opata chromosome 1B probes to characterize the relationship between the accessions (Figure 5a). This highlighted the distinct nature of the ten accessions known to carry the 1RS translocation and confirmed that this introgression was 1BS specific, ending within the 1B centromere (0–133.5 cM covering 34 bins). In addition to the 1RS accessions, we were able to identify eight accessions as distinct from the remaining hexaploids. These accessions carried a unique haplotype covering a significant portion of 1BS and 1BL including the centromeric region (106.3–220.1 cM covering 42 bins). Given the unique 1B haplotype of these accessions, and the similar characteristics that these accessions share with the ten known 1RS accessions, we hypothesized that these might also carry large introgressions on chromosome 1B. To examine the possibility that the Axiom^®^ Array can be used to detect introgressions in the hexaploid wheat genome, we repeated our analysis using the 1266 markers from 7D, which in some lines is known to carry introgressions (Burt *et al*., 2011). This analysis identified ten accessions as having a distinct genotype spanning 38 cM on 7DL (Figure 5b).

## Discussion

We have developed a high‐density wheat genotyping array using the Affymetrix Axiom^®^ platform. This Axiom^®^ HD Wheat Genotyping Array, which is available as a commercial product (Affymetrix product IDs 550491 and 550492 for the two component arrays; http://www.affymetrix.com/support/technical/datasheets/axiom_wheat_hd_genotyping_array_datasheet.pdf), contains 819 571 exome‐captured SNP sequences derived from hexaploid wheat accessions, including both elite and landrace accessions, diploid and tetraploid progenitor accessions and wheat relatives.

A major problem with comparing sequences from a range of species is the difficulty in identifying orthologous sequences leading to the generation of a large number of putative SNPs that cannot be validated. To minimize this problem, we used a NimbleGen hexaploid wheat exome capture array such that only likely orthologous sequences were collected and screened for SNPs (Winfield *et al*., 2012). Based upon the screening conditions employed, we were able to convert 89% of our putative SNPs to probes suitable for the Axiom^®^ Array.

We have shown that the SNPs on the Axiom^®^ Array that could be assigned to IWGSC contigs are evenly distributed across wheat's 21 chromosomes. However, the majority of contigs (81.5%) contained two or more SNPs with some containing as many as 73. The reasons for this skewed distribution are unclear, for although larger contigs tended to contain more SNPs, this alone is not sufficient to account for the large differences in SNP frequency between the contigs. The complexity of the wheat genome and its large proportion of non‐coding sequences is one possible reason for the biased distribution of polymorphic SNPs in the contigs (Gupta *et al*., 2008; Voss‐Fels *et al*., 2015). Further detailed investigation will be necessary to de‐convolute the effects of gene density, polymorphism rate and contig size on SNP density.

Screening the Axiom^®^ Array with 475 accessions resulted in 546 299 (66.7%) ‘useful SNP probes’ (i.e. SNPs that fall into one of the three categories described in Experimental procedures) being called. As expected, the majority of useful probes on the array were polymorphic (any marker for which there is more than one genotype called; a single individual with a distinct genotype is called a polymorphism) between elite hexaploid accessions and wheat relatives. We identified 112 723 polymorphic markers in the hexaploid accessions. Of these, 16 092 (14.2%) were scored as codominant (genotypes scored as AA or BB) rather than dominant probes (scored as either AA and AB or BB and AB). A further 7005 (6.2%) probes were scored as partially codominant (scored as AA or BB with a subset of accessions having an AB call). Codominant and partially codominant markers are extremely useful for generating genetic maps from F2 populations and for tracking introduced genomic fragments in breeding lines (Mammadov *et al*., 2012).

Although we only included eight tetraploid accessions in our screening, 59 079 SNPs were found to be polymorphic between them. This relatively high number suggests that a considerable amount of diversity exists within the tetraploid genepool as has been indicated by Ren *et al*. (2013); using the Axiom^®^ Array, or a smaller derivative, it should be possible to screen large collections of tetraploid lines. Of the 59 079 SNPs that were polymorphic between the eight tetraploid lines, 35 943 were also polymorphic within the hexaploid accessions. These may be useful in future breeding programmes involving the two species.

Polymorphic SNPs were mapped in three populations, Avalon × Cadenza and Savannah × Rialto (UK standard reference populations) and Synthetic × Opata (standard International Triticeae Mapping Initiative population). As expected for the UK populations, the number of markers that mapped to the D genome was considerably lower than those mapping to either the A or B genomes (Akhunov *et al*., 2010). This was not the case for the Synthetic × Opata population; a larger number of markers mapped with a more even distribution between the three genomes (Sorrels *et al*., 2011). However, this greater level of polymorphism comes at a price as markers polymorphic on the Synthetic × Opata population were of limited value when used to screen elite breeding lines. For instance, the average minor allele frequency of the D genome markers from the Synthetic × Opata population was lower (0.1204) than that for SNPs on either Avalon × Cadenza (0.2216) or Savannah × Rialto (0.2946). This result highlights the drawback of using wide crosses to generate molecular makers; although more markers may be generated, many may not be polymorphic on material used in breeding programmes. However, with the wider use of synthetic lines in breeding (reviewed by Li *et al*., 2014), this problem might resolve itself.

Our goal was to generate a genotyping platform capable of characterizing both wheat and its relatives. The Axiom^®^ Array is capable of doing this. For instance, the Axiom^®^ Array was able to separate the D genome progenitor lines into two distinct groups. Lines from the Far East (Kyrgystan and China), which were exclusively *Ae. tauschii* ssp. *tauschii*, formed one group, while lines from the Near East (Armenia, Iran and the west of Turkmenistan), including the three *strangulata* lines, formed a second group (Figure 4b): this is of interest as it is thought that the D genome of hexaploid wheat is derived from this subspecies (Dvorak *et al*., 1998). Similarly, the array was able to separate the AB tetraploid accessions according to subspecies. As these polymorphic SNP probes were able to discriminate between all of the lines used (Figure 4c), this subset of probes may be useful in the generation of a tetraploid‐specific array.

The Axiom^®^ Array also discriminated subgroups among the ABD hexaploid accessions (Figure 4a). The spring and winter wheats clustered separately. The accessions from the Watkins Collection were more similar to the spring accessions than they were to the winter accessions. This agrees with the study by Wingen *et al*. (2014) which suggests that 86% of accessions in the Watkins collection have a spring growth habit. In addition, ten accessions, two spring wheats and eight winter wheats all of which carry the IRS translocation from rye were identified as being distinct. To examine this further, we used the 2306 chromosome 1B markers on the Synthetic × Opata map. These mapped the rye introgression to the short arm of 1B and confirmed that the translocation did not extend beyond the centromere (Figure 5a). An additional eight lines appeared to carry a novel haplotype covering a significant proportion of 1B including the region containing the centromere (Figure 5a). Of these, seven were known to be related via the common progenitor line Cadenza. Our results suggest that Cadenza carries genetic material on 1B distinct from the majority of hexaploid accessions and therefore possibly derived from introgressed material. The eighth line, Batis, is not known to be related to Cadenza, and it is interesting to note that the 1B haplotype for this accession, while being distinct from the remaining hexaploid accessions, is also distinct from Cadenza‐derived accessions and hence represents a novel introgression within the hexaploid accessions examined.

We next investigated whether the Axiom^®^ Array was capable of identifying introgressed material in the hexaploid genome even when it is not from species used to generate the array. Firstly, we examined the array for SNPs previously identified from a species not used in our original design. For this, we used the SNPs identified by Tiwari *et al*. (2014) from chromosome 5M of *Ae. geniculata*. A BLASTN screen of the 104 5M flanking sequences against the 819 571 probes on the array indicated that 48 were present, and of these, 36 were also polymorphic between hexaploid accessions and wheat relatives (Table S8). In addition, ten accessions screened on the array (Azzerti, Battalion, Bermude, Boregar, Lynx, Oratorio, Renan, Revelation, Skyfall and Tuerkis) were known to carry the *Ae. ventricosa* introgression containing the eye spot resistance gene *Pch1* (Doussinault *et al*., 1983; Worland *et al*., 1988). Using the 1266 markers from chromosome 7D of the Synthetic × Opata map, we mapped the *Ae. ventricosa* introgression to the long arm of 7D (Figure 5b). Our analysis showed that the ten accessions fell into two groups depending on the size of the introgression: six lines; Battalion, Boregar, Lynx, Renan, Revelation and Skyfall had the introgression from 456.8 to 556.8 cM, a region containing 76 SNP markers organized into 18 bins, while the other four had a smaller introgression (551.7–556.8 cM, a region containing 20 markers in 4 bins), a result that extends the work previously reported by Burt and Nicholson (2011). Examination of the long arm of chromosome 7D also indicated that a further 14 accessions (Apogee, Adhoc, Altigo, Biscay, Cellule, Duxford, Exotic, Fiorello, Humber, Mercato, Panorama, Premio, Santiago and Solstice) carry a telomeric introgression but that this is distinct from the *Pch1 Ae. ventricosa* introgression. Both of these analyses clearly indicated that the Axiom^®^ Array has utility even when used to screen genotypes and species not used in the original array design.

In conclusion, the development of the Axiom^®^ HD Wheat Genotyping Array, which is capable of characterizing a range of wheat‐related species, together with the associated automated genotyping call algorithms, high‐density maps and public database will provide the wheat community with a valuable resource for the characterization and breeding of hexaploid and tetraploid wheat. In addition, the availability of a high‐density array capable of tracking the introgression and subsequent fate of chromosomal fragments from a range of wheat relatives could revolutionize wheat breeding and ensure that such introgressions can be utilized with greater efficiency by targeting further breeding to reduce the size of the fragments and hence reduce linkage drag.

## Experimental procedures

### Plant material

The accessions grown for DNA extraction (listed in Table S2) were grown in peat‐based soil in pots and maintained in a glasshouse at 15–25 °C with 16‐h light, 8‐h dark. Leaf tissue was harvested from 6‐week‐old plants, immediately frozen on liquid nitrogen and then stored at −20 °C prior to nucleic acid extraction. Genomic DNA was prepared from leaf tissue using a phenol–chloroform extraction method (Sambrook *et al*., 1989). Genomic DNA samples were treated with RNase‐A (New England Biolabs UK Ltd., Hitchin, UK), according to the manufacturer's instructions and purified using the QiaQuick PCR purification kit (QIAGEN Ltd., Manchester, UK).

### Exome capture and next‐generation sequencing

Exome capture and next‐generation sequencing were performed on 43 accessions (Table S1) according to Winfield *et al*. (2012). The pipeline removes all within‐variety (homoeologous) SNPS which make up the vast majority of variants in hexaploid wheat.

Sequencing data can be downloaded from the NCBI Sequence Read Archive (SRA) from the Axiom^®^ 820 Wheat Array Data study PRJNA286098, accession SRP059312 (accession numbers for all the lines included in study are in Table S9).

### SNP discovery

After preprocessing of reads to remove adapter sequences, the data were submitted to a custom pipeline (Winfield *et al*., 2012). Putative SNPs, together with their flanking sequences, were processed using the Affymetrix design protocol for the Axiom^®^ platform to generate SNP probes for array.

### Sequence alignment

Sequence alignment was carried out using Exonerate version 2.2.0 with parameters—model ungapped, per cent 0 and bestn 3.

### Genotyping

The Axiom^®^ Wheat HD Genotyping Arrays was used to genotype 475 samples (Table S2) using the Affymetrix GeneTitan^®^ system according to the procedure described by Affymetrix (Axiom^®^ 2.0 Assay Manual Workflow User Guide Rev3). Allele calling was carried out using the Affymetrix proprietary software packages Affymetrix Power Tools (APT) and SNPolisher^™^ (http://www.affymetrix.com/estore/partners_programs/programs/developer/tools/devnettools.affx). A custom software pipeline ADAP (Axiom^®^ Data Analysis Pipeline) was written in perl to simplify the data analysis, following the Axiom^®^ Best Practices Genotyping Workflow (http://media.affymetrix.com/support/downloads/manuals/axiom_genotyping_solution_analysis_guide.pdf). A variant call rate threshold of 80% was used instead of the default value (97%) to account for the lower call rates typically obtained from hybridizing wheat relatives and progenitors to the array. The apt‐probeset‐genotype program within Affymetrix Power Tools determines genotype calls from Affymetrix SNP microarrays. Following this, the SNPolisher R package calculates SNP performance metrics, such as call rate, cluster separation and deviation from expected cluster position. It then classifies the SNPs into performance categories. These categories were as follows: (i) PHR, which were codominant and polymorphic, with at least two examples of the minor allele; (ii) NMH, which were polymorphic and dominant, with two clusters observed; (iii) OTV, which had four clusters, one representing a null allele; (iv) MHR, which were monomorphic; (v) CRBT, where SNP call rate was below threshold but other cluster properties were above threshold; and (vi) Other, where one or more cluster properties were below threshold.

### Genetic map construction

Individuals from three doubled‐haploid mapping populations were genotyped with the Axiom^®^ HD Wheat Genotyping Array. From the Avalon × Cadenza population, 130 lines were genotyped, 64 lines from the Savannah × Rialto population and 60 lines from the Synthetic × Opata population. For each population, markers with more than 20% missing data were removed and markers were binned based on their pattern of segregation in each respective population using the BIN function in ICIMapping V.3.3 (Meng *et al*., 2015). Markers were placed into the same bin if the correlation coefficient between them was one, and therefore, the recombination frequency between them was estimated as 0. Following binning, all markers which displayed a unique pattern of segregation and did not fall into a bin were removed. Markers that shared their pattern of segregation with at least one other were retained, and one marker was chosen to represent each bin, either one with the least amount of missing data, or in the case where the percentage of missing data was equal, at random.

Markers were tested for significant segregation distortion using a chi‐square test and those with significant distortion (*P* < 0.05) were removed. Markers were sorted into groups in MapDisto version 1.7.5 Beta 4 (Lorieux, 2012) with a LOD score of six and recombination fraction of 0.3 using the Kosambi mapping function (Kosambi, 1943). Groups were ordered with the seriation algorithm. These were exported and assigned to chromosomes using information from an Exonerate alignment to the IWGSC wheat survey sequence (The International Wheat Genome sequencing Consortium, 2014), genotype scores from the Kansas deletion lines (Endo and Gill, 1996) and genotype scores from wheat nullisomic/tetrasomic lines (Devos *et al*., 1999). Where chromosomes were split into multiple linkage groups, these were re‐formed into a single linkage group and re‐ordered. Marker order within each chromosome group was optimized with an iterative process of rippling the marker order using a window size of five markers and checking for inversions until the best possible order was found.

The long and short arm of each chromosome was identified from the IWGSC wheat survey sequence (The International Wheat Genome Sequencing Consortium, 2014), and groups were orientated to have the short arm above the long arm. Following map construction, the binned markers were integrated back into the map.

### Generating a wheat consensus map

Where there was agreement, all markers were assigned to a ‘consensus chromosome’ based on information from the genetic maps. In the case of conflicts between two or all the maps, information from the nullisomic lines, the Kansas deletion lines and the IWGSC survey sequences was used to assign markers to a consensus chromosome.

The consensus map was generated using the R package ‘LPMerge’ (Endelman and Plomion, 2014). No weighting was given to the component maps. In the case of duplicates, a marker was retained if its position in the consensus map matched the previously defined ‘consensus chromosome’ and its duplicate was removed. Where there was no ‘consensus chromosome’ designation, one of the duplicates was removed at random.

### Dimensionality reduction

The relationship between the lines was determined by calculating a similarity matrix for all the lines (Table S7). This was calculated as number of markers shared by any two lines divided by total number of markers for the two lines; markers that had missing calls for either of the lines were not used to estimate similarity. The matrices were imported into R and used to create principal coordinate plots using the classic MDS method, cmdscale.

Graphical genotype visualization and hierarchical clustering were performed using Spotfire software (TIBCO, Boston, MA), using default parameters. Prior to importing into Spotfire, genotype scores were coded for each locus as: 1 = least common genotype score; 2 = second most common genotype score; and 3 = most common genotype score.

## Supporting information


**Table S1** Accessions subjected to NimbleGen targeted re‐sequencing.

**Table S2** Accessions assayed on the Axiom HD Wheat Genotyping Array.

**Table S3** Sample Calls Rates.

**Table S4** Summary information for genetic maps.

**Table S5** Genetic maps and consensus.

**Table S6** Conflicting markers.

**Table S7** Similarity matrix for all accessions.

**Table S8** Sequences present in the Tiwari *et al*. study.

**Table S9** Accession numbers for the NCBI Sequence Read Archive.
