# Supplementary material for: High‐density SNP genotyping array for hexaploid wheat and its secondary and tertiary gene pool
Source: Plant Biotechnol J. 2015 Oct 15;14(5):1195–206. doi: 10.1111/pbi.12485 (PMC4950041; doi:10.1111/pbi.12485)
Supplement: Supplementary file 1 — Table S1 Accessions subjected to NimbleGen targeted re‐sequencing. [file PBI-14-1195-s004.docx]

**Supplementary File 1**

Accessions subjected to NimbleGen targeted re-sequencing (listed by ploidy level and then genome). The number of sequences generated is presented along with the number of reads mapped to the reference sequence reported in Winfield *et al*. (2012). The named tetraploid lines belong to *T. turgidum* (syn. *T. durum*). Species names are those proposed by van Slageren, 1994.

| **Species or lines (synonym)** | **Genome** | **Status** | **Trimmed reads** | **Mapped reads** |
| --- | --- | --- | --- | --- |
|  |  |  | **(x million)** | **(x million)** |
| **Diploids** |  |  |  |  |
| *T. monococcum* ssp. *aegilopoides* (*T. urartu*) | A | Progenitor | 11.5 | 3.6 |
| *Ae. tauschii* 008 | D | Progenitor | 20.4 | 5.4 |
| *Ae. tauschii* 232 | D | Progenitor | 34.4 | 12.4 |
| *Ae. tauschii* 320 | D | Progenitor | 14.2 | 5.2 |
| *Ae. tauschii* 336 | D | Progenitor | 54.6 | 14.4 |
| *Ae. tauschii* 392 | D | Progenitor | 17.7 | 4.1 |
| *Ae. tauschii* 414 | D | Progenitor | 18 | 4.1 |
| *Ae. tauschii* 2220007 | D | Progenitor | 9.3 | 3.5 |
| *Thinopyrum elongatum* | E | Wild relative | 1.1 | 0.3 |
| *Thinopyrum bessarabicum* | J | Wild relative | 2.1 | 0.5 |
| *Secale cereale* | R | Wild relative | 1.7 | 0.4 |
| *Ae. speltoides* | S* | Progenitor | 6 | 1.6 |
| *Ae. markgrafii* (*Ae. caudata*) | T | Wild relative | 6 | 1.3 |
| *Amblyopyrum muticum* (*Ae. mutica*) | T | Wild relative | 0.3 | 0.1 |
|  |  |  |  |  |
| **Tetraploid accessions** |  |  |  |  |
| *T. turgidum* ssp. *durum* Creso | AB | Progenitor | 20.3 | 7 |
| *T. turgidum* ssp. *durum* KU37 | AB | Progenitor | 23.9 | 4.3 |
| *T. turgidum* ssp.  *dicoccoides* TTD140 | AB | Progenitor | 6.6 | 2.1 |
| *T. timopheevii* | AG | Wild relative | 17.5 | 5.7 |
| *Ae. peregrina* (*Ae. variabilis*) | SU | Wild relative | 22.6 | 3.4 |
|  |  |  |  |  |
| **Hexaploid accessions** |  |  |  |  |
| Alchemy | ABD | Breeder line | 29.8 | 11.3 |
| Apogee | ABD | Breeder line | 15.8 | 6.6 |
| Avalon | ABD | Breeder line | 45.2 | 12.1 |
| Cadenza | ABD | Breeder line | 32.4 | 6.3 |
| Chinese Spring L42 | ABD | Breeder line | 11.3 | 2.8 |
| Hereward | ABD | Breeder line | 27.8 | 10.2 |
| Highbury | ABD | Breeder line | 15.9 | 5.5 |
| Paragon type 1 (AA) | ABD | Breeder line | 44.6 | 16.4 |
| Paragon type 2 (KJE) | ABD | Breeder line | 6.4 | 2.3 |
| Pavon 76 | ABD | Breeder line | 17.2 | 3.2 |
| Rialto | ABD | Breeder line | 50.9 | 14.3 |
| Robigus | ABD | Breeder line | 23.2 | 4.4 |
| Savannah | ABD | Breeder line | 74 | 19.5 |
| Xi19 | ABD | Breeder line | 23.8 | 9.1 |
| Watkins Line 34 | ABD | Watkins | 10.2 | 3.4 |
| Watkins Line 126 | ABD | Watkins | 11.4 | 2.4 |
| Watkins Line 141 | ABD | Watkins | 4.9 | 1.7 |
| Watkins Line 199 | ABD | Watkins | 24.6 | 6 |
| Watkins Line 209 | ABD | Watkins | 74.6 | 26.1 |
| Watkins Line 292 | ABD | Watkins | 12.7 | 4.4 |
| Watkins Line 352 | ABD | Watkins | 8.8 | 3 |
| Watkins Line 468 | ABD | Watkins | 7.8 | 2.7 |
| Watkins Line 729 | ABD | Watkins | 11.1 | 3.6 |
| *Thinopyrum intermedium* | SJJ | Wild relative | 10.8 | 1.6 |
|  |  |  |  |  |
| **Decaploid accessions** |  |  |  |  |
| *Thinopyrum ponticum* | JJJJsJs | Wild relative | 16.3 | 2.9 |

Species names used are essentially those proposed in van Slageren, M.W. (1994)  Wild wheats: a monograph of *Aegilops* L. and *Amblyopyrum* (Jaub. & Spach) Eig (Poaceae).  ICARDA, Aleppo, Syria.

The genome designation of *Thinopyrum* species used in this table are based on the paper: Chen, Q., Conner, R.L., Laroche, A. And Thomas (1998) Genome analysis of *Thinopyrum intermedium* and *Thinopyrum ponticum* using genomic in situ hybridization. *Genome* **41**, 580 – 586.

*The S genome of *Ae. speltoides*  is very closely related, but not identical, to the wheat B genome (Akhunov, E.D., Alina R Akhunova, A.R., Anderson, O.D., Anderson, J.A., Blake, N., Clegg, M.T., Coleman-Derr, D., Conley, E.J., Crossman, C.C., Deal, K.R., Dubcovsky, J., Gill, B.J., Gu, Y.Q., Hadam, J., Heo, H., Huo, N., Lazo, G.R., Luo, M-C., Ma1, Y.Q., Matthews, D.E., McGuire, P.E., Morrel, P.L., Qualset, C.O., Renfro, J., Tabanao, D., Talbert, L.E., Tian, C., Toleno, D.M., Warburton,M.L., You, F.M., Zhang, W. and Dvorak, J. (2010) Nucleotide diversity maps reveal variation in diversity among wheat genomes and chromosomes. *BMC Genomics* **11**, 702 (<http://www.biomedcentral.com/1471-2164/11/702>)
