# Supplementary material for: High‐density SNP genotyping array for hexaploid wheat and its secondary and tertiary gene pool
Source: Plant Biotechnol J. 2015 Oct 15;14(5):1195–206. doi: 10.1111/pbi.12485 (PMC4950041; doi:10.1111/pbi.12485)
Supplement: Supplementary file 3 — Table S3 Sample Calls Rates. [file PBI-14-1195-s006.docx]

Supplementary Table 3: Average sample call rates for the different accessions used to screen the Axiom Array (sorted by call rate).

|  | Number of lines | Average call rate |
| --- | --- | --- |
| Wheat relatives | 10 | 85.82190 |
| *Ae. tauschii* | 14 | 92.34620 |
| *T. turgidum* | 8 | 94.34003 |
| Deletion lines | 52 | 98.31611 |
| Watkins landraces | 27 | 98.88635 |
| Mapping populations | 256 | 98.92175 |
| Elite cultivars | 108 | 99.18991 |
| All lines | 475 | 98.40772 |
