# Supplementary material for: High‐density SNP genotyping array for hexaploid wheat and its secondary and tertiary gene pool
Source: Plant Biotechnol J. 2015 Oct 15;14(5):1195–206. doi: 10.1111/pbi.12485 (PMC4950041; doi:10.1111/pbi.12485)
Supplement: Supplementary file 4 — Table S4 Summary information for genetic maps. [file PBI-14-1195-s008.docx]

**Supplementary File 4**

**Mapping Results**

**Avalon x Cadenza**

Length, in centiMorgans, for the 21 chromosomes of the Avalon x Cadenza doubled haploid population mapped with SNPs from the Axiom 820K HD genotyping array

|  | A | B | D | Total |
| --- | --- | --- | --- | --- |
| 1 | 155.65 | 178.10 | 142.08 | 475.84 |
| 2 | 185.11 | 211.32 | 172.94 | 569.38 |
| 3 | 197.96 | 260.65 | 148.39 | 607.01 |
| 4 | 195.77 | 121.65 | 7.20 | 324.62 |
| 5 | 235.40 | 215.14 | 105.04 | 555.58 |
| 6 | 175.85 | 165.99 | 162.77 | 504.61 |
| 7 | 215.93 | 255.24 | 154.39 | 625.56 |
| Total | 1361.67 | 1408.10 | 892.82 | 3662.60 |

The B genome contained the most markers and the D genome contained the fewest, at 9, 609 and 2, 448 respectively (Table 2).

Number of single nucleotide polymorphism markers which mapped to each of the 21 chromosomes of the Avalon x Cadenza DH population

|  | A | B | D | Total |
| --- | --- | --- | --- | --- |
| 1 | 988 | 2379 | 896 | 4263 |
| 2 | 873 | 1709 | 506 | 3088 |
| 3 | 697 | 1051 | 152 | 1900 |
| 4 | 905 | 599 | 55 | 1559 |
| 5 | 802 | 1191 | 425 | 2418 |
| 6 | 1459 | 1557 | 143 | 3159 |
| 7 | 1161 | 1123 | 271 | 2555 |
| Total | 6885 | 9609 | 2448 | 18942 |

Genetic map of the Avalon x Cadenza DH population. Groups are oriented with the short arm above the long arm.

**Savannah x Rialto**

Length, in centiMorgans, for the 21 chromosomes of the Savannah x Rialto doubled haploid population mapped with SNPs from the Axiom 820K HD genotyping array

|  | A | B | D | Total |
| --- | --- | --- | --- | --- |
| 1 | 182.07 | 152.68 | 90.5 | 425.25 |
| 2 | 37.85 | 193.16 | 109.12 | 340.13 |
| 3 | 166.33 | 136.82 | 162.59 | 465.74 |
| 4 | 187.87 | 47.49 | 7.85 | 243.21 |
| 5 | 249.59 | 271.36 | 128.07 | 649.02 |
| 6 | 166.55 | 132.48 | 56.3 | 355.33 |
| 7 | 179.58 | 72.11 | 130.84 | 382.53 |
| Total | 1169.84 | 1006.1 | 685.27 | 2861.21 |

Number of single nucleotide polymorphism markers which mapped to each of the 21 chromosomes of the Savannah x Rialto DH population

|  | A | B | D | Total |
| --- | --- | --- | --- | --- |
| 1 | 1353 | 885 | 471 | 2709 |
| 2 | 487 | 2876 | 61 | 3424 |
| 3 | 630 | 1037 | 368 | 2035 |
| 4 | 242 | 225 | 86 | 553 |
| 5 | 1048 | 985 | 413 | 2446 |
| 6 | 1146 | 1771 | 187 | 3104 |
| 7 | 965 | 559 | 244 | 1768 |
| Total | 5871 | 8338 | 1830 | 16039 |

Genetic map of the Savannah x Rialto DH population. Groups are oriented with the short arm above the long arm, with the exception of chromosome 6D, whcih falls into 2 groups

**Synthetic x Opata**

Length, in centiMorgans, for the 21 chromosomes of the Synthetic x Opata doubled haploid population mapped with SNPs from the Axiom 820K HD genotyping array

|  | A | B | D | Total |
| --- | --- | --- | --- | --- |
| 1 | 362.19 | 396.79 | 389.91 | 1148.89 |
| 2 | 315.78 | 490.42 | 386.95 | 1193.15 |
| 3 | 372.50 | 313.19 | 358.76 | 1044.44 |
| 4 | 375.87 | 238.82 | 198.23 | 812.91 |
| 5 | 383.17 | 384.99 | 434.56 | 1202.72 |
| 6 | 324.50 | 283.19 | 327.87 | 935.55 |
| 7 | 400.94 | 449.75 | 556.79 | 1407.48 |
| Total | 2534.94 | 2557.14 | 2653.07 | 7745.15 |

Number of single nucleotide polymorphism markers which mapped to each of the 21 chromosomes of the Synthetic x Opata DH population

|  | A | B | D | Total |
| --- | --- | --- | --- | --- |
| 1 | 1371 | 2306 | 979 | 4656 |
| 2 | 1285 | 2368 | 1168 | 4821 |
| 3 | 1144 | 1787 | 2010 | 4941 |
| 4 | 1518 | 1240 | 703 | 3461 |
| 5 | 1022 | 2138 | 1519 | 4679 |
| 6 | 1565 | 1739 | 1192 | 4496 |
| 7 | 1378 | 2110 | 1266 | 4754 |
| Total | 9283 | 13688 | 8837 | 31808 |

Genetic map of the Synthetic x Opata DH population. Groups are oriented with the short arm above the long arm.

**Consensus map**

56,505 markers mapped to one of the 21 wheat chromosomes in a consensus map (Table 1).

The total map length was 3,739.2 cM, with an average of 178 cM per chromosome (Table 2)

Number of markers mapped to each chromosome in the consensus map

|  | A | B | D | Total |
| --- | --- | --- | --- | --- |
| 1 | 2938 | 4303 | 2077 | 9318 |
| 2 | 2451 | 5967 | 1599 | 10017 |
| 3 | 2083 | 3207 | 2344 | 7634 |
| 4 | 2358 | 1756 | 813 | 4927 |
| 5 | 2348 | 3844 | 2019 | 8211 |
| 6 | 3129 | 4090 | 1397 | 8616 |
| 7 | 2851 | 3251 | 1680 | 7782 |
| Total | 18158 | 26418 | 11929 | 56505 |

Length of each chromosome (cM) of the consensus map

|  | A | B | D | Total |
| --- | --- | --- | --- | --- |
| 1 | 182.1 | 182.4 | 151.29 | 515.71 |
| 2 | 204 | 217 | 177.47 | 598.42 |
| 3 | 136.1 | 234.6 | 234.87 | 605.54 |
| 4 | 75.68 | 76.67 | 162.07 | 314.42 |
| 5 | 221.4 | 208.8 | 167.57 | 597.71 |
| 6 | 189.4 | 166 | 167.78 | 523.17 |
| 7 | 231.6 | 279.3 | 73.34 | 584.26 |
| Total | 1240 | 1365 | 1134.4 | 3739.23 |

A consensus map of 56,505 single nucleotide polymorphism markers from the Affymetrix Axiom HD wheat genotyping array. The consensus map was generated from three individual componenent maps from the Avalon x Cadenza, Savannah x Rialto and Synthetic x Opata doubled haploid mapping populations.
