# Supplementary material for: High‐density SNP genotyping array for hexaploid wheat and its secondary and tertiary gene pool
Source: Plant Biotechnol J. 2015 Oct 15;14(5):1195–206. doi: 10.1111/pbi.12485 (PMC4950041; doi:10.1111/pbi.12485)
Supplement: Supplementary file 9 — Table S9 Accession numbers for the NCBI Sequence Read Archive. [file PBI-14-1195-s005.docx]

**Accession numbers for data used to generate array probes**

| **Type** | **Alias** | **Accession** |
| --- | --- | --- |
| STUDY | PRJNA286098 | SRP059312 |
| SAMPLE | *Triticum urartu* | SRS957607 |
| EXPERIMENT | *Triticum urartu* | SRX1054856 |
| RUN | *Triticum urartu* | SRR2058072 |
| SAMPLE | Ent-008 | SRS957627 |
| EXPERIMENT | *Aegilops tauschii* 008 | SRX1054915 |
| RUN | *Aegilops tauschii* 008 | SRR2058111 |
| SAMPLE | Ent-336 | SRS957628 |
| EXPERIMENT | *Aegilops tauschii* 336 | SRX1054932 |
| RUN | *Aegilops tauschii* 336 | SRR2058125 |
| SAMPLE | Ent-392 | SRS957629 |
| EXPERIMENT | *Aegilops tauschii* 392 | SRX1054937 |
| RUN | *Aegilops tauschii* 392 | SRR2058132 |
| SAMPLE | Ent-414 | SRS957630 |
| EXPERIMENT | *Aegilops tauschii* 414 | SRX1054942 |
| RUN | *Aegilops tauschii* 414 | SRR2058138 |
| SAMPLE | JIC_2220007 | SRS957647 |
| EXPERIMENT | *Aegilops tauschii* 2220007 | SRX1054959 |
| RUN | *Aegilops tauschii* 2220007 | SRR2058175 |
| SAMPLE | *Thinopyrum elongatium* | SRS957648 |
| EXPERIMENT | *Thinopyrum elongatum* | SRX1054960 |
| RUN | *Thinopyrum elongatum* | SRR2058177 |
| SAMPLE | *Thinopyrum bessarabicum* | SRS957650 |
| EXPERIMENT | *Thinopyrum bessarabicum* | SRX1054962 |
| RUN | *Thinopyrum bessarabicum* | SRR2058179 |
| SAMPLE | Rye | SRS957651 |
| EXPERIMENT | Rye | SRX1054963 |
| RUN | Rye | SRR2058180 |
| RUN | Rye 2 | SRR2060066 |
| SAMPLE | *Aegilops speltoides* | SRS957652 |
| EXPERIMENT | *Aegilops speltoides* | SRX1054965 |
| RUN | *Aegilops speltoides* | SRR2058181 |
| SAMPLE | [*Aegilops caudata*](http://trace.ncbi.nlm.nih.gov/Traces/sra_sub/?subid=472579&amp;action=show:SAMPLE&amp;acc=SRS957653&amp;noheader=1#0) | SRS957653 |
| EXPERIMENT | [*Aegilops markgrafii*](http://trace.ncbi.nlm.nih.gov/Traces/sra_sub/?subid=472579&amp;action=show:EXPERIMENT&amp;acc=SRX1054966&amp;noheader=1#0) | SRX1054966 |
| RUN | [*Aegilops markgrafii* (*Aegilops caudata*)](http://trace.ncbi.nlm.nih.gov/Traces/sra_sub/?subid=472579&amp;action=show:RUN&amp;acc=SRR2058182&amp;noheader=1#0) | SRR2058182 |
| SAMPLE | [Ae_mutica](http://trace.ncbi.nlm.nih.gov/Traces/sra_sub/?subid=472579&amp;action=show:SAMPLE&amp;acc=SRS957654&amp;noheader=1#0) | SRS957654 |
| EXPERIMENT | [*Amblyopyrum muticum*](http://trace.ncbi.nlm.nih.gov/Traces/sra_sub/?subid=472579&amp;action=show:EXPERIMENT&amp;acc=SRX1054967&amp;noheader=1#0) | SRX1054967 |
| RUN | [*Amblyopyrum muticum* (*Aegilops mutica*)](http://trace.ncbi.nlm.nih.gov/Traces/sra_sub/?subid=472579&amp;action=show:RUN&amp;acc=SRR2058183&amp;noheader=1#0) | SRR2058183 |
| SAMPLE | [Creso](http://trace.ncbi.nlm.nih.gov/Traces/sra_sub/?subid=472579&amp;action=show:SAMPLE&amp;acc=SRS957656&amp;noheader=1#0) | SRS957656 |
| EXPERIMENT | [*Triticum turgidum* ssp. *durum* Creso](http://trace.ncbi.nlm.nih.gov/Traces/sra_sub/?subid=472579&amp;action=show:EXPERIMENT&amp;acc=SRX1054969&amp;noheader=1#0) | SRX1054969 |
| RUN | [Creso](http://trace.ncbi.nlm.nih.gov/Traces/sra_sub/?subid=472579&amp;action=show:RUN&amp;acc=SRR2058186&amp;noheader=1#0) | SRR2058186 |
| SAMPLE | [KU37](http://trace.ncbi.nlm.nih.gov/Traces/sra_sub/?subid=472579&amp;action=show:SAMPLE&amp;acc=SRS957726&amp;noheader=1#0) | SRS957726 |
| EXPERIMENT | [*Triticum turgidum* ssp. *durum* KU37](http://trace.ncbi.nlm.nih.gov/Traces/sra_sub/?subid=472579&amp;action=show:EXPERIMENT&amp;acc=SRX1055041&amp;noheader=1#0) | SRX1055041 |
| RUN | [KU37](http://trace.ncbi.nlm.nih.gov/Traces/sra_sub/?subid=472579&amp;action=show:RUN&amp;acc=SRR2058328&amp;noheader=1#0) | SRR2058328 |
| SAMPLE | [KJE_A_Ttd140](http://trace.ncbi.nlm.nih.gov/Traces/sra_sub/?subid=472579&amp;action=show:SAMPLE&amp;acc=SRS957732&amp;noheader=1#0) | SRS957732 |
| EXPERIMENT | [*Triticum turgidum* ssp. *dicoccoides* TTD140](http://trace.ncbi.nlm.nih.gov/Traces/sra_sub/?subid=472579&amp;action=show:EXPERIMENT&amp;acc=SRX1055044&amp;noheader=1#0) | SRX1055044 |
| RUN | [TTD140](http://trace.ncbi.nlm.nih.gov/Traces/sra_sub/?subid=472579&amp;action=show:RUN&amp;acc=SRR2058331&amp;noheader=1#0) | SRR2058331 |
| SAMPLE | [*T_timoph*](http://trace.ncbi.nlm.nih.gov/Traces/sra_sub/?subid=472579&amp;action=show:SAMPLE&amp;acc=SRS957767&amp;noheader=1#0)*eevii* | SRS957767 |
| EXPERIMENT | [*Triticum timopheevii*](http://trace.ncbi.nlm.nih.gov/Traces/sra_sub/?subid=472579&amp;action=show:EXPERIMENT&amp;acc=SRX1055045&amp;noheader=1#0) | SRX1055045 |
| RUN | [*Triticum timopheevii*](http://trace.ncbi.nlm.nih.gov/Traces/sra_sub/?subid=472579&amp;action=show:RUN&amp;acc=SRR2058332&amp;noheader=1#0) | SRR2058332 |
| SAMPLE | [KJE_B2_Ae.tauschii](http://trace.ncbi.nlm.nih.gov/Traces/sra_sub/?subid=472579&amp;action=show:SAMPLE&amp;acc=SRS958878&amp;noheader=1#0) | SRS958878 |
| EXPERIMENT | [*Aegilops tauschii* 232](http://trace.ncbi.nlm.nih.gov/Traces/sra_sub/?subid=472579&amp;action=show:EXPERIMENT&amp;acc=SRX1056211&amp;noheader=1#0) | SRX1056211 |
| RUN | [*Aegilops tauschii* 232](http://trace.ncbi.nlm.nih.gov/Traces/sra_sub/?subid=472579&amp;action=show:RUN&amp;acc=SRR2060070&amp;noheader=1#0) | SRR2060070 |
| SAMPLE | [KJE_C2_Ae.tech](http://trace.ncbi.nlm.nih.gov/Traces/sra_sub/?subid=472579&amp;action=show:SAMPLE&amp;acc=SRS958883&amp;noheader=1#0) | SRS958883 |
| EXPERIMENT | [*Aegilops tauschii* 320](http://trace.ncbi.nlm.nih.gov/Traces/sra_sub/?subid=472579&amp;action=show:EXPERIMENT&amp;acc=SRX1056217&amp;noheader=1#0) | SRX1056217 |
| RUN | [*Aegilops tauschii* 320](http://trace.ncbi.nlm.nih.gov/Traces/sra_sub/?subid=472579&amp;action=show:RUN&amp;acc=SRR2060072&amp;noheader=1#0) | SRR2060072 |
| SAMPLE | [*Ae.variabilis*](http://trace.ncbi.nlm.nih.gov/Traces/sra_sub/?subid=472579&amp;action=show:SAMPLE&amp;acc=SRS958885&amp;noheader=1#0) | SRS958885 |
| EXPERIMENT | [*Aegilops peregrina* (*Aegilops variabilis*)](http://trace.ncbi.nlm.nih.gov/Traces/sra_sub/?subid=472579&amp;action=show:EXPERIMENT&amp;acc=SRX1056218&amp;noheader=1#0) | SRX1056218 |
| RUN | [*Aegilops peregrina* (*Aegilops variabilis*)](http://trace.ncbi.nlm.nih.gov/Traces/sra_sub/?subid=472579&amp;action=show:RUN&amp;acc=SRR2060073&amp;noheader=1#0) | SRR2060073 |
| SAMPLE | [ukAlchemy](http://trace.ncbi.nlm.nih.gov/Traces/sra_sub/?subid=472579&amp;action=show:SAMPLE&amp;acc=SRS958886&amp;noheader=1#0) | SRS958886 |
| EXPERIMENT | [Alchemy](http://trace.ncbi.nlm.nih.gov/Traces/sra_sub/?subid=472579&amp;action=show:EXPERIMENT&amp;acc=SRX1056219&amp;noheader=1#0) | SRX1056219 |
| RUN | [Alchemy](http://trace.ncbi.nlm.nih.gov/Traces/sra_sub/?subid=472579&amp;action=show:RUN&amp;acc=SRR2060074&amp;noheader=1#0) | SRR2060074 |
| EXPERIMENT | [Alchemy2](http://trace.ncbi.nlm.nih.gov/Traces/sra_sub/?subid=472579&amp;action=show:EXPERIMENT&amp;acc=SRX1056322&amp;noheader=1#0) | SRX1056322 |
| RUN | [Alchemy2](http://trace.ncbi.nlm.nih.gov/Traces/sra_sub/?subid=472579&amp;action=show:RUN&amp;acc=SRR2060186&amp;noheader=1#0) | SRR2060186 |
| SAMPLE | [Apogee](http://trace.ncbi.nlm.nih.gov/Traces/sra_sub/?subid=472579&amp;action=show:SAMPLE&amp;acc=SRS958887&amp;noheader=1#0) | SRS958887 |
| EXPERIMENT | [Apogee](http://trace.ncbi.nlm.nih.gov/Traces/sra_sub/?subid=472579&amp;action=show:EXPERIMENT&amp;acc=SRX1056220&amp;noheader=1#0) | SRX1056220 |
| RUN | [Apogee](http://trace.ncbi.nlm.nih.gov/Traces/sra_sub/?subid=472579&amp;action=show:RUN&amp;acc=SRR2060075&amp;noheader=1#0) | SRR2060075 |
| SAMPLE | [Avalon](http://trace.ncbi.nlm.nih.gov/Traces/sra_sub/?subid=472579&amp;action=show:SAMPLE&amp;acc=SRS958888&amp;noheader=1#0) | SRS958888 |
| EXPERIMENT | [Avalon](http://trace.ncbi.nlm.nih.gov/Traces/sra_sub/?subid=472579&amp;action=show:EXPERIMENT&amp;acc=SRX1056221&amp;noheader=1#0) | SRX1056221 |
| RUN | [Avalon](http://trace.ncbi.nlm.nih.gov/Traces/sra_sub/?subid=472579&amp;action=show:RUN&amp;acc=SRR2060076&amp;noheader=1#0) | SRR2060076 |
| SAMPLE | [Cadenza](http://trace.ncbi.nlm.nih.gov/Traces/sra_sub/?subid=472579&amp;action=show:SAMPLE&amp;acc=SRS958889&amp;noheader=1#0) | SRS958889 |
| EXPERIMENT | [Cadenza](http://trace.ncbi.nlm.nih.gov/Traces/sra_sub/?subid=472579&amp;action=show:EXPERIMENT&amp;acc=SRX1056222&amp;noheader=1#0) | SRX1056222 |
| RUN | [Cadenza](http://trace.ncbi.nlm.nih.gov/Traces/sra_sub/?subid=472579&amp;action=show:RUN&amp;acc=SRR2060077&amp;noheader=1#0) | SRR2060077 |
| SAMPLE | [ukXi19](http://trace.ncbi.nlm.nih.gov/Traces/sra_sub/?subid=472579&amp;action=show:SAMPLE&amp;acc=SRS959455&amp;noheader=1#0) | SRS959455 |
| EXPERIMENT | [Xi19](http://trace.ncbi.nlm.nih.gov/Traces/sra_sub/?subid=472579&amp;action=show:EXPERIMENT&amp;acc=SRX1056939&amp;noheader=1#0) | SRX1056939 |
| RUN | [Xi19_1](http://trace.ncbi.nlm.nih.gov/Traces/sra_sub/?subid=472579&amp;action=show:RUN&amp;acc=SRR2060973&amp;noheader=1#0) | SRR2060973 |
| EXPERIMENT | [Xi19_2](http://trace.ncbi.nlm.nih.gov/Traces/sra_sub/?subid=472579&amp;action=show:EXPERIMENT&amp;acc=SRX1056942&amp;noheader=1#0) | SRX1056942 |
| RUN | [Xi19_2](http://trace.ncbi.nlm.nih.gov/Traces/sra_sub/?subid=472579&amp;action=show:RUN&amp;acc=SRR2060974&amp;noheader=1#0) | SRR2060974 |
| SAMPLE | [ChineseSpringL42](http://trace.ncbi.nlm.nih.gov/Traces/sra_sub/?subid=472579&amp;action=show:SAMPLE&amp;acc=SRS959459&amp;noheader=1#0) | SRS959459 |
| EXPERIMENT | [Chinese Spring L42](http://trace.ncbi.nlm.nih.gov/Traces/sra_sub/?subid=472579&amp;action=show:EXPERIMENT&amp;acc=SRX1056944&amp;noheader=1#0) | SRX1056944 |
| RUN | [Chinese Spring L42](http://trace.ncbi.nlm.nih.gov/Traces/sra_sub/?subid=472579&amp;action=show:RUN&amp;acc=SRR2060981&amp;noheader=1#0) | SRR2060981 |
| SAMPLE | [Highbury](http://trace.ncbi.nlm.nih.gov/Traces/sra_sub/?subid=472579&amp;action=show:SAMPLE&amp;acc=SRS959460&amp;noheader=1#0) | SRS959460 |
| EXPERIMENT | [Highbury](http://trace.ncbi.nlm.nih.gov/Traces/sra_sub/?subid=472579&amp;action=show:EXPERIMENT&amp;acc=SRX1056945&amp;noheader=1#0) | SRX1056945 |
| RUN | [Highbury](http://trace.ncbi.nlm.nih.gov/Traces/sra_sub/?subid=472579&amp;action=show:RUN&amp;acc=SRR2060983&amp;noheader=1#0) | SRR2060983 |
| SAMPLE | [AA_Paragon](http://trace.ncbi.nlm.nih.gov/Traces/sra_sub/?subid=472579&amp;action=show:SAMPLE&amp;acc=SRS959461&amp;noheader=1#0) | SRS959461 |
| EXPERIMENT | [AA_Paragon](http://trace.ncbi.nlm.nih.gov/Traces/sra_sub/?subid=472579&amp;action=show:EXPERIMENT&amp;acc=SRX1056946&amp;noheader=1#0) | SRX1056946 |
| RUN | [AA_Paragon](http://trace.ncbi.nlm.nih.gov/Traces/sra_sub/?subid=472579&amp;action=show:RUN&amp;acc=SRR2060984&amp;noheader=1#0) | SRR2060984 |
| SAMPLE | [KJE_Paragon](http://trace.ncbi.nlm.nih.gov/Traces/sra_sub/?subid=472579&amp;action=show:SAMPLE&amp;acc=SRS959462&amp;noheader=1#0) | SRS959462 |
| EXPERIMENT | [KJE_Paragon](http://trace.ncbi.nlm.nih.gov/Traces/sra_sub/?subid=472579&amp;action=show:EXPERIMENT&amp;acc=SRX1056947&amp;noheader=1#0) | SRX1056947 |
| RUN | [KJE_Paragon](http://trace.ncbi.nlm.nih.gov/Traces/sra_sub/?subid=472579&amp;action=show:RUN&amp;acc=SRR2060985&amp;noheader=1#0) | SRR2060985 |
| SAMPLE | [Pavon76](http://trace.ncbi.nlm.nih.gov/Traces/sra_sub/?subid=472579&amp;action=show:SAMPLE&amp;acc=SRS959463&amp;noheader=1#0) | SRS959463 |
| EXPERIMENT | [Pavon76](http://trace.ncbi.nlm.nih.gov/Traces/sra_sub/?subid=472579&amp;action=show:EXPERIMENT&amp;acc=SRX1056948&amp;noheader=1#0) | SRX1056948 |
| RUN | [Pavon76](http://trace.ncbi.nlm.nih.gov/Traces/sra_sub/?subid=472579&amp;action=show:RUN&amp;acc=SRR2060986&amp;noheader=1#0) | SRR2060986 |
| SAMPLE | [Rialto](http://trace.ncbi.nlm.nih.gov/Traces/sra_sub/?subid=472579&amp;action=show:SAMPLE&amp;acc=SRS959465&amp;noheader=1#0) | SRS959465 |
| EXPERIMENT | [Rialto](http://trace.ncbi.nlm.nih.gov/Traces/sra_sub/?subid=472579&amp;action=show:EXPERIMENT&amp;acc=SRX1056951&amp;noheader=1#0) | SRX1056951 |
| RUN | [Rialto](http://trace.ncbi.nlm.nih.gov/Traces/sra_sub/?subid=472579&amp;action=show:RUN&amp;acc=SRR2060987&amp;noheader=1#0) | SRR2060987 |
| SAMPLE | [ukRobigus](http://trace.ncbi.nlm.nih.gov/Traces/sra_sub/?subid=472579&amp;action=show:SAMPLE&amp;acc=SRS959467&amp;noheader=1#0) | SRS959467 |
| EXPERIMENT | [Robigus](http://trace.ncbi.nlm.nih.gov/Traces/sra_sub/?subid=472579&amp;action=show:EXPERIMENT&amp;acc=SRX1056953&amp;noheader=1#0) | SRX1056953 |
| RUN | [Robigus](http://trace.ncbi.nlm.nih.gov/Traces/sra_sub/?subid=472579&amp;action=show:RUN&amp;acc=SRR2060991&amp;noheader=1#0) | SRR2060991 |
| SAMPLE | [Savannah](http://trace.ncbi.nlm.nih.gov/Traces/sra_sub/?subid=472579&amp;action=show:SAMPLE&amp;acc=SRS959469&amp;noheader=1#0) | SRS959469 |
| EXPERIMENT | [Savannah](http://trace.ncbi.nlm.nih.gov/Traces/sra_sub/?subid=472579&amp;action=show:EXPERIMENT&amp;acc=SRX1056955&amp;noheader=1#0) | SRX1056955 |
| RUN | [Savannah](http://trace.ncbi.nlm.nih.gov/Traces/sra_sub/?subid=472579&amp;action=show:RUN&amp;acc=SRR2060992&amp;noheader=1#0) | SRR2060992 |
| SAMPLE | [Watkins34](http://trace.ncbi.nlm.nih.gov/Traces/sra_sub/?subid=472579&amp;action=show:SAMPLE&amp;acc=SRS959470&amp;noheader=1#0) | SRS959470 |
| EXPERIMENT | [Watkins34](http://trace.ncbi.nlm.nih.gov/Traces/sra_sub/?subid=472579&amp;action=show:EXPERIMENT&amp;acc=SRX1056956&amp;noheader=1#0) | SRX1056956 |
| RUN | [Watkins34](http://trace.ncbi.nlm.nih.gov/Traces/sra_sub/?subid=472579&amp;action=show:RUN&amp;acc=SRR2060996&amp;noheader=1#0) | SRR2060996 |
| SAMPLE | [Watkins126](http://trace.ncbi.nlm.nih.gov/Traces/sra_sub/?subid=472579&amp;action=show:SAMPLE&amp;acc=SRS959476&amp;noheader=1#0) | SRS959476 |
| EXPERIMENT | [Watkins126](http://trace.ncbi.nlm.nih.gov/Traces/sra_sub/?subid=472579&amp;action=show:EXPERIMENT&amp;acc=SRX1056971&amp;noheader=1#0) | SRX1056971 |
| RUN | [Watkins126](http://trace.ncbi.nlm.nih.gov/Traces/sra_sub/?subid=472579&amp;action=show:RUN&amp;acc=SRR2061019&amp;noheader=1#0) | SRR2061019 |
| SAMPLE | [*Thinopyrum ponticum*](http://trace.ncbi.nlm.nih.gov/Traces/sra_sub/?subid=472579&amp;action=show:SAMPLE&amp;acc=SRS959477&amp;noheader=1#0) | SRS959477 |
| EXPERIMENT | [*Thinopyrum ponticum*](http://trace.ncbi.nlm.nih.gov/Traces/sra_sub/?subid=472579&amp;action=show:EXPERIMENT&amp;acc=SRX1056972&amp;noheader=1#0) | SRX1056972 |
| RUN | [*Thinopyrum ponticum*](http://trace.ncbi.nlm.nih.gov/Traces/sra_sub/?subid=472579&amp;action=show:RUN&amp;acc=SRR2061020&amp;noheader=1#0) | SRR2061020 |
| SAMPLE | [Watkins141](http://trace.ncbi.nlm.nih.gov/Traces/sra_sub/?subid=472579&amp;action=show:SAMPLE&amp;acc=SRS959481&amp;noheader=1#0) | SRS959481 |
| EXPERIMENT | [Watkins141](http://trace.ncbi.nlm.nih.gov/Traces/sra_sub/?subid=472579&amp;action=show:EXPERIMENT&amp;acc=SRX1056976&amp;noheader=1#0) | SRX1056976 |
| RUN | [Watkins141](http://trace.ncbi.nlm.nih.gov/Traces/sra_sub/?subid=472579&amp;action=show:RUN&amp;acc=SRR2061038&amp;noheader=1#0) | SRR2061038 |
| SAMPLE | [*Thinopyrum intermedium*](http://trace.ncbi.nlm.nih.gov/Traces/sra_sub/?subid=472579&amp;action=show:SAMPLE&amp;acc=SRS959485&amp;noheader=1#0) | SRS959485 |
| EXPERIMENT | [*Thinopyrum intermedium*](http://trace.ncbi.nlm.nih.gov/Traces/sra_sub/?subid=472579&amp;action=show:EXPERIMENT&amp;acc=SRX1056982&amp;noheader=1#0) | SRX1056982 |
| RUN | [*Thinopyrum intermedium*](http://trace.ncbi.nlm.nih.gov/Traces/sra_sub/?subid=472579&amp;action=show:RUN&amp;acc=SRR2061044&amp;noheader=1#0) | SRR2061044 |
| SAMPLE | [Watkins_199](http://trace.ncbi.nlm.nih.gov/Traces/sra_sub/?subid=472579&amp;action=show:SAMPLE&amp;acc=SRS959487&amp;noheader=1#0) | SRS959487 |
| EXPERIMENT | [Watkins_199](http://trace.ncbi.nlm.nih.gov/Traces/sra_sub/?subid=472579&amp;action=show:EXPERIMENT&amp;acc=SRX1056985&amp;noheader=1#0) | SRX1056985 |
| RUN | [Watkins_199](http://trace.ncbi.nlm.nih.gov/Traces/sra_sub/?subid=472579&amp;action=show:RUN&amp;acc=SRR2061047&amp;noheader=1#0) | SRR2061047 |
| SAMPLE | [AA_Watkins209](http://trace.ncbi.nlm.nih.gov/Traces/sra_sub/?subid=472579&amp;action=show:SAMPLE&amp;acc=SRS959488&amp;noheader=1#0) | SRS959488 |
| EXPERIMENT | [Watkins209](http://trace.ncbi.nlm.nih.gov/Traces/sra_sub/?subid=472579&amp;action=show:EXPERIMENT&amp;acc=SRX1056986&amp;noheader=1#0) | SRX1056986 |
| RUN | [Watkins209](http://trace.ncbi.nlm.nih.gov/Traces/sra_sub/?subid=472579&amp;action=show:RUN&amp;acc=SRR2061048&amp;noheader=1#0) | SRR2061048 |
| SAMPLE | [AA_WatkinsW292](http://trace.ncbi.nlm.nih.gov/Traces/sra_sub/?subid=472579&amp;action=show:SAMPLE&amp;acc=SRS959494&amp;noheader=1#0) | SRS959494 |
| EXPERIMENT | [Watkins292](http://trace.ncbi.nlm.nih.gov/Traces/sra_sub/?subid=472579&amp;action=show:EXPERIMENT&amp;acc=SRX1056993&amp;noheader=1#0) | SRX1056993 |
| RUN | [Watkins292](http://trace.ncbi.nlm.nih.gov/Traces/sra_sub/?subid=472579&amp;action=show:RUN&amp;acc=SRR2061054&amp;noheader=1#0) | SRR2061054 |
| SAMPLE | [Watkins352](http://trace.ncbi.nlm.nih.gov/Traces/sra_sub/?subid=472579&amp;action=show:SAMPLE&amp;acc=SRS959675&amp;noheader=1#0) | SRS959675 |
| EXPERIMENT | [Watkins352](http://trace.ncbi.nlm.nih.gov/Traces/sra_sub/?subid=472579&amp;action=show:EXPERIMENT&amp;acc=SRX1057162&amp;noheader=1#0) | SRX1057162 |
| RUN | [Watkins352](http://trace.ncbi.nlm.nih.gov/Traces/sra_sub/?subid=472579&amp;action=show:RUN&amp;acc=SRR2061223&amp;noheader=1#0) | SRR2061223 |
| SAMPLE | [Watkins468](http://trace.ncbi.nlm.nih.gov/Traces/sra_sub/?subid=472579&amp;action=show:SAMPLE&amp;acc=SRS959678&amp;noheader=1#0) | SRS959678 |
| EXPERIMENT | [Watkins468](http://trace.ncbi.nlm.nih.gov/Traces/sra_sub/?subid=472579&amp;action=show:EXPERIMENT&amp;acc=SRX1057165&amp;noheader=1#0) | SRX1057165 |
| RUN | [Watkins468](http://trace.ncbi.nlm.nih.gov/Traces/sra_sub/?subid=472579&amp;action=show:RUN&amp;acc=SRR2061229&amp;noheader=1#0) | SRR2061229 |
| SAMPLE | [Watkins729](http://trace.ncbi.nlm.nih.gov/Traces/sra_sub/?subid=472579&amp;action=show:SAMPLE&amp;acc=SRS959746&amp;noheader=1#0) | SRS959746 |
| EXPERIMENT | [Watkins729](http://trace.ncbi.nlm.nih.gov/Traces/sra_sub/?subid=472579&amp;action=show:EXPERIMENT&amp;acc=SRX1057285&amp;noheader=1#0) | SRX1057285 |
| RUN | [Watkins729](http://trace.ncbi.nlm.nih.gov/Traces/sra_sub/?subid=472579&amp;action=show:RUN&amp;acc=SRR2061355&amp;noheader=1#0) | SRR2061355 |
| SAMPLE | [ukHereward](http://trace.ncbi.nlm.nih.gov/Traces/sra_sub/?subid=472579&amp;action=show:SAMPLE&amp;acc=SRS960682&amp;noheader=1#0) | SRS960682 |
| EXPERIMENT | [Hereward](http://trace.ncbi.nlm.nih.gov/Traces/sra_sub/?subid=472579&amp;action=show:EXPERIMENT&amp;acc=SRX1058313&amp;noheader=1#0) | SRX1058313 |
| RUN | [Hereward](http://trace.ncbi.nlm.nih.gov/Traces/sra_sub/?subid=472579&amp;action=show:RUN&amp;acc=SRR2062442&amp;noheader=1#0) | SRR2062442 |
| EXPERIMENT | [Hereward_2](http://trace.ncbi.nlm.nih.gov/Traces/sra_sub/?subid=472579&amp;action=show:EXPERIMENT&amp;acc=SRX1058316&amp;noheader=1#0) | SRX1058316 |
| RUN | [Hereward_2](http://trace.ncbi.nlm.nih.gov/Traces/sra_sub/?subid=472579&amp;action=show:RUN&amp;acc=SRR2062445&amp;noheader=1#0) | SRR2062445 |
